# Endovascular therapy in iliac artery and lower extremity peripheral arterial diseases

**DOI:** 10.1186/1749-8090-8-S1-P89

**Published:** 2013-09-11

**Authors:** O Tetik, S Surer, Y Besir, O Rodoplu

**Affiliations:** 1 Department of Cardiovascular Surgery, Manisa Celal Bayar University School of Medicine, Manisa, Turkey; 2 Department of Cardiovascular Surgery, Yuksek Ihtisas Training and Research Hospital, Bursa, Turkey; 3 Department of Cardiovascular Surgery, Ataturk Training and Research Hospital, Izmir, Turkey

## Background

Methods of endovascular therapy stent have been increasingly used in addition to conventional surgical approaches in the treatment of peripheral arterial diseases. In this paper we present the endovascular treatment methods that we use in patients with peripheral arterial disease.

## Methods

Twenty-four of patients were treated with endovascular method. Nineteen of the patients had intermittent claudication, 4 had rest pain and one patient with bilateral popliteal artery aneurysms had pain at the back of bilateral knee joints. Twenty patients were male and 3 were female. The mean age was 64,05 (range 40-82 years) years. Nine of the patients who underwent endovascular therapy had a lesion in TASC-A group, 5 had a TASC-B lesion, and TASC-COPD lesions were present in 9 patients. Subintimal balloon angioplasty failed with TASC-C lesions of 2 patients who were excluded from the study. One of these patients underwent urgent infragenual femoropopliteal bypass using a saphenous graft and the other supragenual femoropopliteal bypass using a PTFE graft.

## Results

Bioabsorbable stents, self-expandable nitinol stents, balloon angioplasty, and subintimal balloons were used for treatment. Bioabsorbable stent was implanted in 11 patients. Balloon angioplasty was performed in 3 patients. A self-expandable nitinol stent was deployed in 3 patients. Subintimal balloon angioplasty was performed in 6 patients. An aneurysm repair was performed with stent graft in 1 patient who had bilateral popliteal and right anterior tibial artery aneurysms.

## Conclusions

Urgent success was achieved in all patients. Patient symptoms were markedly improved at the postoperative period and the patients with event-free postoperative clinical course were discharged with complete recovery. No vascular problems were observed during the follow-up of the patients.

